# Diaqua­(1,4,8,11-tetra­aza­cyclo­tetra­deca­ne)nickel(II) fumarate tetra­hydrate

**DOI:** 10.1107/S1600536810020064

**Published:** 2010-06-05

**Authors:** Shao Liang Lim, Chew Hee Ng, Siang Guan Teoh, Wan-Sin Loh, Hoong-Kun Fun

**Affiliations:** a Faculty of Engineering and Science, Universiti Tunku Abdul Rahman, 53300 Kuala Lumpur, Malaysia; b School of Chemical Science, Universiti Sains Malaysia, 11800 USM, Penang, Malaysia; c X-ray Crystallography Unit, School of Physics, Universiti Sains Malaysia, 11800 USM, Penang, Malaysia

## Abstract

The asymmetric unit of the title complex salt, [Ni(C_10_H_24_N_4_)(H_2_O)_2_](C_4_H_2_O_4_)·4H_2_O, comprises half of a nickel(II) complex dication, half of a fumarate dianion and two water mol­ecules. Both the Ni^II^ cation and fumarate anion lie on a crystallographic inversion center. The Ni^II^ ion in the cyclam complex is six-coordinated within a distorted N_4_O_2_ octa­hedral geometry, with the four cyclam N atoms in the equatorial plane and the two water mol­ecules in apical positions. The six-membered metalla ring adopts a chair conformation, whereas the five-membered ring exists in a twisted form. In the crystal packing, inter­molecular O—H⋯O hydrogen bonds between the water molecules and the carboxyl groups of the fumarate anions lead to the formation of layers with *R*_4_^2^(8) ring motifs. Ni^II^ complex cations are sandwiched between two such layers, being held in place by O—H⋯O, N—H⋯O and C—H⋯O hydrogen bonds, consolidating a three-dimensional network.

## Related literature

For the background to and the biological activity of cyclam, see: Kim *et al.* (2006 ▶); Hunter *et al.* (2006 ▶); Gerlach *et al.* (2003 ▶); Paisey & Sadler (2004 ▶). For a related structure, see: Panneerselvam *et al.* (1999 ▶). For puckering parameters, see: Cremer & Pople (1975 ▶). For hydrogen-bond motifs, see: Bernstein *et al.* (1995 ▶). For the stability of the temperature controller used for the data collection, see: Cosier & Glazer (1986 ▶).
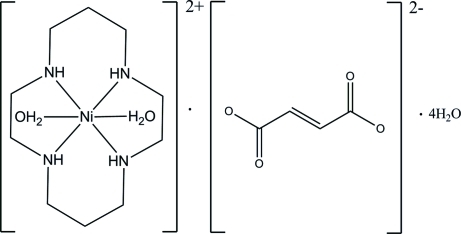


## Experimental

### 

#### Crystal data

[Ni(C_10_H_24_N_4_)(H_2_O)_2_](C_4_H_2_O_4_)·4H_2_O
*M* *_r_* = 481.19
Triclinic, 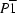

*a* = 6.9913 (5) Å
*b* = 8.8313 (7) Å
*c* = 9.3147 (8) Å
α = 73.165 (2)°
β = 79.207 (2)°
γ = 85.227 (2)°
*V* = 540.47 (7) Å^3^
*Z* = 1
Mo *K*α radiation
μ = 0.95 mm^−1^
*T* = 100 K
0.47 × 0.44 × 0.24 mm

#### Data collection

Bruker APEXII DUO CCD area-detector diffractometer
Absorption correction: multi-scan (*SADABS*; Bruker, 2009 ▶) *T*_min_ = 0.665, *T*_max_ = 0.805
12800 measured reflections
4295 independent reflections
4219 reflections with *I* > 2σ(*I*)
*R*_int_ = 0.017

#### Refinement

*R*[*F* ^2^ > 2σ(*F* ^2^)] = 0.035
*wR*(*F* ^2^) = 0.133
*S* = 1.30
4295 reflections
142 parameters
H atoms treated by a mixture of independent and constrained refinement
Δρ_max_ = 1.27 e Å^−3^
Δρ_min_ = −1.18 e Å^−3^

### 

Data collection: *APEX2* (Bruker, 2009 ▶); cell refinement: *SAINT* (Bruker, 2009 ▶); data reduction: *SAINT*; program(s) used to solve structure: *SHELXTL* (Sheldrick, 2008 ▶); program(s) used to refine structure: *SHELXTL*; molecular graphics: *SHELXTL*; software used to prepare material for publication: *SHELXTL* and *PLATON* (Spek, 2009 ▶).

## Supplementary Material

Crystal structure: contains datablocks global, I. DOI: 10.1107/S1600536810020064/tk2677sup1.cif

Structure factors: contains datablocks I. DOI: 10.1107/S1600536810020064/tk2677Isup2.hkl

Additional supplementary materials:  crystallographic information; 3D view; checkCIF report

## Figures and Tables

**Table 1:** Hydrogen-bond geometry (Å, °)

*D*—H⋯*A*	*D*—H	H⋯*A*	*D*⋯*A*	*D*—H⋯*A*
O1*W*—H1*W*1⋯O3*W*	0.85	2.17	2.8047 (14)	131
O2*W*—H1*W*2⋯O2^i^	0.85	1.98	2.7026 (14)	142
O2*W*—H2*W*2⋯O2^ii^	0.85	1.91	2.7000 (15)	154
O3*W*—H1*W*3⋯O1^iii^	0.85	1.96	2.7633 (14)	157
O3*W*—H2*W*3⋯O1	0.85	2.06	2.7968 (14)	144
N1—H1*N*1⋯O2*W*^iv^	0.88 (2)	2.19 (2)	3.0153 (15)	154 (2)
N2—H1*N*2⋯O3*W*^iv^	0.90 (2)	2.25 (2)	3.0769 (15)	153 (2)
C3—H3*B*⋯O1^v^	0.97	2.60	3.3850 (18)	138

Symmetry codes: (i) 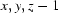; (ii) 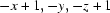; (iii) 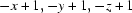; (iv) 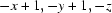; (v) 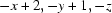.

**Fig. 1.**
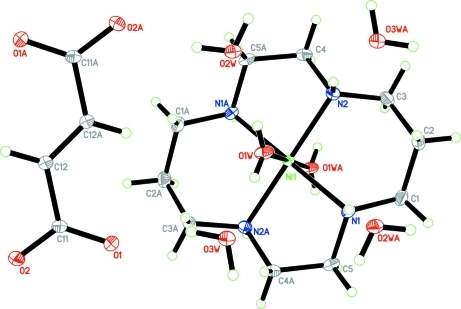
The molecular structure of the title complex, showing 50% probability displacement ellipsoids and the atom-numbering scheme. Symmetry-related atoms of the NiII complex ion and fumarate anion are generated by the symmetry codes -x+2, -y+1, -z and -x+1, -y, -z+1, respectively.

**Fig. 2.**
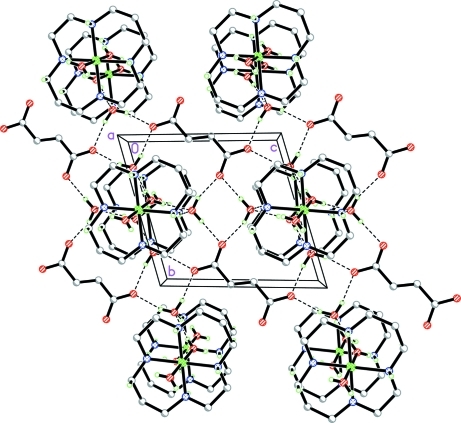
The crystal packing of the title compound, viewed approximately along the a axis, showing the three-dimensional network. H atoms not involved in the intermolecular interactions (dashed lines) have been omitted for clarity.

## supplementary crystallographic information

### Comment

The antiviral properties of cyclam (1,4,8,11-tetraazacyclotetradecane) have stimulated interest in metal complexes of this ligand (Kim *et al.*, 2006). Besides its antiviral property, [Ni(cyclam)(OAc)_2_] also has protein recognition potential (Hunter *et al.*, 2006). Amongst the metal ions investigated, coordination of Ni^II^ to cyclam rings bridged by 1,4-dimethylene(phenylene) was reported to result in greatest enhancement of its antiviral property (Gerlach *et al.*, 2003). However, the rate of complexation of Ni^II^ to cyclam is the poorest compared to Cu^II^, Zn^II^ and Co^II^ (Paisey *et al.*, 2004). In this paper, we report the crystal structure of the title compound, obtained by the reaction of a nickel(II) salt, cyclam and sodium fumarate.

The title compound, Fig. 1, consists of one nickel(II) complex cation, one fumarate anion and four water molecules. Both Ni^II^ ion and fumarate anion lie on a crystallographic inversion center, generated by the symmetry codes -x+2, -y+1, -z and -x+1, -y, -z+1, respectively. The Ni^II^ complex of cyclam has six-coordination in a distorted octahedral geometry, with the four ligand N atoms (N1/N2/N1A/N2A) almost coplanar with the Ni^II^ ion and the two water molecules (O1W & O1WA) in apical positions. The six-membered ring (Ni1/N1/C1–C3/N2) exists in a chair conformation with the puckering parameters (Cremer & Pople, 1975) Q = 0.5900 (14) Å; Θ = 9.05 (13)° and φ = 192.1 (9)°. In the five-membered ring, Ni1/N1/C5/C4A/N2A is twisted about the C5–C4A bond with the puckering parameters (Cremer & Pople, 1975) Q = 0.4382 (14) Å and φ = 271.34 (14)°. This structure is comparable to a closely related structure (Panneerselvam *et al.*, 1999).

In the crystal packing (Fig. 2), intermolecular O_water_—H···O_carboxylate_, hydrogen bonds (Table 1) link with the carboxyl groups of the fumarate anions into a two-dimensional layers with *R*^2^_4_(8) ring motifs (Bernstein *et al.*, 1995). The Ni^II^ complex cations are linked to these layers by O_aquo_—H···O_water_, N_amine_—H···O_water_, C3—H3B···O_carboxylate_ hydrogen bonds (Table 1) to form a three-dimensional network.

### Experimental

Nickel chloride hexahydrate (0.24 g, 1 mmol), cyclam (0.22 g, 1 mmol) and sodium fumarate (0.16 g, 1 mmol) were dissolved in water and heated overnight in a water bath at 313 K. Purple crystals were obtained from the yellow solution.

### Refinement

N-bound H atoms (H1N1 & H2N1) were located from the difference map and refined freely. The O-bound H atoms were also located in a difference map but were then fixed in their as found positions with *U*_iso_(H)  = 1.5 *U*_eq_(O). The remaining H atoms were positioned geometrically and refined using a riding model, with *U*_iso_(H) = 1.2 or 1.5 *U*_eq_(C) [C–H = 0.93 or 0.97 Å; N–H = 0.85 (2) to 0.86 (2) Å; O–H = 0.8482 to 0.8537 Å]. The maximum and minimum residual electron density peaks of 1.300 and -1.178 eÅ^-3^, respectively, were located 0.36 Å and 0.94 Å from the N1 and Ni1 atoms, respectively.

### Crystal data

**Table:**

[Ni(C_10_H_24_N_4_)(H_2_O)_2_](C_4_H_2_O_4_)·4H_2_O	*Z* = 1
*M**_r_* = 481.19	*F*(000) = 258
Triclinic, *P*1	*D*_x_ = 1.478 Mg m^−^^3^
Hall symbol: -P 1	Mo *K*α radiation, λ = 0.71073 Å
*a* = 6.9913 (5) Å	Cell parameters from 9982 reflections
*b* = 8.8313 (7) Å	θ = 3.8–35.1°
*c* = 9.3147 (8) Å	µ = 0.95 mm^−^^1^
α = 73.165 (2)°	*T* = 100 K
β = 79.207 (2)°	Block, purple
γ = 85.227 (2)°	0.47 × 0.44 × 0.24 mm
*V* = 540.47 (7) Å^3^	

### Data collection

**Table:**

Bruker APEXII DUO CCD area-detector diffractometer	4295 independent reflections
Radiation source: fine-focus sealed tube	4219 reflections with *I* > 2σ(*I*)
graphite	*R*_int_ = 0.017
φ and ω scans	θ_max_ = 34.0°, θ_min_ = 3.0°
Absorption correction: multi-scan (*SADABS*; Bruker, 2009)	*h* = −10→10
*T*_min_ = 0.665, *T*_max_ = 0.805	*k* = −13→13
12800 measured reflections	*l* = −14→14

### Refinement

**Table:**

Refinement on *F*^2^	Secondary atom site location: difference Fourier map
Least-squares matrix: full	Hydrogen site location: inferred from neighbouring sites
*R*[*F*^2^ > 2σ(*F*^2^)] = 0.035	H atoms treated by a mixture of independent and constrained refinement
*wR*(*F*^2^) = 0.133	*w* = 1/[σ^2^(*F*_o_^2^) + (0.0892*P*)^2^ + 0.062*P*] where *P* = (*F*_o_^2^ + 2*F*_c_^2^)/3
*S* = 1.30	(Δ/σ)_max_ < 0.001
4295 reflections	Δρ_max_ = 1.27 e Å^−^^3^
142 parameters	Δρ_min_ = −1.18 e Å^−^^3^
0 restraints	Extinction correction: *SHELXTL* (Sheldrick, 2008), Fc^*^=kFc[1+0.001xFc^2^λ^3^/sin(2θ)]^-1/4^
Primary atom site location: structure-invariant direct methods	Extinction coefficient: 0.75 (4)

### Special details

**Table:**

Experimental. The crystal was placed in the cold stream of an Oxford Cryosystems Cobra open-flow nitrogen cryostat (Cosier & Glazer, 1986) operating at 100.0 (1) K.
Geometry. All esds (except the esd in the dihedral angle between two l.s. planes) are estimated using the full covariance matrix. The cell esds are taken into account individually in the estimation of esds in distances, angles and torsion angles; correlations between esds in cell parameters are only used when they are defined by crystal symmetry. An approximate (isotropic) treatment of cell esds is used for estimating esds involving l.s. planes.
Refinement. Refinement of F^2^ against ALL reflections. The weighted R-factor wR and goodness of fit S are based on F^2^, conventional R-factors R are based on F, with F set to zero for negative F^2^. The threshold expression of F^2^ > 2sigma(F^2^) is used only for calculating R-factors(gt) etc. and is not relevant to the choice of reflections for refinement. R-factors based on F^2^ are statistically about twice as large as those based on F, and R- factors based on ALL data will be even larger.

### Fractional atomic coordinates and isotropic or equivalent isotropic displacement parameters (Å^2^)

**Table:**

	*x*	*y*	*z*	*U*_iso_*/*U*_eq_	
Ni1	1.0000	0.5000	0.0000	0.00889 (11)	
O1W	0.70136 (13)	0.43457 (11)	0.07879 (11)	0.01471 (17)	
H1W1	0.5819	0.4284	0.1199	0.022*	
H2W1	0.7631	0.3578	0.1313	0.022*	
N1	0.90487 (15)	0.73294 (12)	−0.04325 (12)	0.01220 (18)	
N2	0.97429 (15)	0.48188 (12)	−0.21216 (12)	0.01229 (18)	
C1	0.97192 (19)	0.83674 (14)	−0.19756 (15)	0.0160 (2)	
H1A	1.1117	0.8476	−0.2125	0.019*	
H1B	0.9108	0.9411	−0.2068	0.019*	
C2	0.9231 (2)	0.77130 (15)	−0.32091 (15)	0.0188 (2)	
H2A	0.7853	0.7497	−0.2980	0.023*	
H2B	0.9461	0.8528	−0.4173	0.023*	
C3	1.0368 (2)	0.62134 (16)	−0.34023 (14)	0.0168 (2)	
H3A	1.0178	0.6032	−0.4344	0.020*	
H3B	1.1746	0.6357	−0.3474	0.020*	
C4	1.08394 (18)	0.33580 (15)	−0.22827 (14)	0.0154 (2)	
H4A	1.2220	0.3563	−0.2578	0.018*	
H4B	1.0424	0.3011	−0.3070	0.018*	
C5	0.95195 (18)	0.79227 (14)	0.07821 (15)	0.0144 (2)	
H5A	0.8713	0.8855	0.0855	0.017*	
H5B	1.0873	0.8217	0.0545	0.017*	
O1	0.52695 (16)	0.26163 (11)	0.55707 (11)	0.01632 (18)	
O2	0.52311 (19)	0.08177 (12)	0.78098 (11)	0.0224 (2)	
C11	0.51455 (17)	0.12258 (13)	0.64080 (13)	0.0124 (2)	
C12	0.48943 (17)	−0.00891 (13)	0.57443 (12)	0.0121 (2)	
H12A	0.4574	−0.1080	0.6408	0.014*	
O2W	0.52940 (14)	0.20503 (11)	0.01387 (11)	0.01441 (17)	
H1W2	0.5831	0.1689	−0.0597	0.022*	
H2W2	0.5251	0.1297	0.0954	0.022*	
O3W	0.42354 (14)	0.50357 (11)	0.31238 (11)	0.01463 (18)	
H1W3	0.4170	0.5898	0.3362	0.022*	
H2W3	0.3993	0.4291	0.3948	0.022*	
H1N1	0.779 (3)	0.719 (3)	−0.034 (3)	0.018 (5)*	
H1N2	0.849 (3)	0.463 (3)	−0.210 (3)	0.015 (5)*	

### Atomic displacement parameters (Å^2^)

**Table:**

	*U*^11^	*U*^22^	*U*^33^	*U*^12^	*U*^13^	*U*^23^
Ni1	0.00948 (13)	0.00806 (13)	0.00991 (13)	−0.00033 (7)	−0.00201 (7)	−0.00347 (8)
O1W	0.0114 (4)	0.0161 (4)	0.0187 (4)	−0.0023 (3)	−0.0003 (3)	−0.0089 (3)
N1	0.0115 (4)	0.0103 (4)	0.0151 (4)	−0.0006 (3)	−0.0026 (3)	−0.0038 (3)
N2	0.0125 (4)	0.0134 (4)	0.0120 (4)	−0.0008 (3)	−0.0025 (3)	−0.0047 (3)
C1	0.0183 (5)	0.0108 (4)	0.0175 (5)	−0.0020 (4)	−0.0043 (4)	−0.0006 (4)
C2	0.0237 (6)	0.0158 (5)	0.0163 (5)	−0.0013 (4)	−0.0087 (4)	−0.0003 (4)
C3	0.0212 (5)	0.0180 (5)	0.0111 (4)	−0.0034 (4)	−0.0030 (4)	−0.0029 (4)
C4	0.0170 (5)	0.0161 (5)	0.0153 (5)	−0.0003 (4)	−0.0011 (4)	−0.0089 (4)
C5	0.0143 (5)	0.0117 (4)	0.0193 (5)	−0.0008 (3)	−0.0023 (4)	−0.0080 (4)
O1	0.0251 (5)	0.0105 (4)	0.0131 (4)	−0.0020 (3)	−0.0028 (3)	−0.0029 (3)
O2	0.0434 (6)	0.0145 (4)	0.0110 (4)	−0.0018 (4)	−0.0080 (4)	−0.0041 (3)
C11	0.0158 (5)	0.0113 (4)	0.0110 (4)	−0.0002 (3)	−0.0017 (3)	−0.0050 (3)
C12	0.0153 (5)	0.0107 (4)	0.0105 (4)	−0.0004 (3)	−0.0019 (3)	−0.0037 (3)
O2W	0.0174 (4)	0.0131 (4)	0.0146 (4)	−0.0020 (3)	−0.0032 (3)	−0.0061 (3)
O3W	0.0175 (4)	0.0144 (4)	0.0134 (4)	−0.0017 (3)	−0.0034 (3)	−0.0052 (3)

### Geometric parameters (Å, °)

**Table:**

Ni1—N1^i^	2.0564 (10)	C2—H2B	0.9700
Ni1—N1	2.0565 (10)	C3—H3A	0.9700
Ni1—N2	2.0699 (10)	C3—H3B	0.9700
Ni1—N2^i^	2.0699 (10)	C4—C5^i^	1.5153 (18)
Ni1—O1W	2.1478 (9)	C4—H4A	0.9700
Ni1—O1W^i^	2.1478 (9)	C4—H4B	0.9700
O1W—H1W1	0.8499	C5—C4^i^	1.5153 (18)
O1W—H2W1	0.8506	C5—H5A	0.9700
N1—C5	1.4747 (16)	C5—H5B	0.9700
N1—C1	1.4772 (17)	O1—C11	1.2496 (14)
N1—H1N1	0.88 (2)	O2—C11	1.2615 (14)
N2—C3	1.4745 (16)	C11—C12	1.5007 (16)
N2—C4	1.4761 (16)	C12—C12^ii^	1.330 (2)
N2—H1N2	0.90 (2)	C12—H12A	0.9300
C1—C2	1.5279 (19)	O2W—H1W2	0.8501
C1—H1A	0.9700	O2W—H2W2	0.8496
C1—H1B	0.9700	O3W—H1W3	0.8482
C2—C3	1.5253 (19)	O3W—H2W3	0.8537
C2—H2A	0.9700		
			
N1^i^—Ni1—N1	180.0	C2—C1—H1B	109.2
N1^i^—Ni1—N2	85.49 (4)	H1A—C1—H1B	107.9
N1—Ni1—N2	94.51 (4)	C3—C2—C1	115.74 (11)
N1^i^—Ni1—N2^i^	94.51 (4)	C3—C2—H2A	108.3
N1—Ni1—N2^i^	85.49 (4)	C1—C2—H2A	108.3
N2—Ni1—N2^i^	179.999 (1)	C3—C2—H2B	108.3
N1^i^—Ni1—O1W	91.94 (4)	C1—C2—H2B	108.3
N1—Ni1—O1W	88.06 (4)	H2A—C2—H2B	107.4
N2—Ni1—O1W	88.73 (4)	N2—C3—C2	111.79 (10)
N2^i^—Ni1—O1W	91.27 (4)	N2—C3—H3A	109.3
N1^i^—Ni1—O1W^i^	88.06 (4)	C2—C3—H3A	109.3
N1—Ni1—O1W^i^	91.94 (4)	N2—C3—H3B	109.3
N2—Ni1—O1W^i^	91.27 (4)	C2—C3—H3B	109.3
N2^i^—Ni1—O1W^i^	88.73 (4)	H3A—C3—H3B	107.9
O1W—Ni1—O1W^i^	180.0	N2—C4—C5^i^	109.50 (10)
Ni1—O1W—H1W1	165.2	N2—C4—H4A	109.8
Ni1—O1W—H2W1	77.0	C5^i^—C4—H4A	109.8
H1W1—O1W—H2W1	107.7	N2—C4—H4B	109.8
C5—N1—C1	113.05 (9)	C5^i^—C4—H4B	109.8
C5—N1—Ni1	106.83 (7)	H4A—C4—H4B	108.2
C1—N1—Ni1	116.66 (8)	N1—C5—C4^i^	109.30 (9)
C5—N1—H1N1	112.6 (16)	N1—C5—H5A	109.8
C1—N1—H1N1	108.2 (16)	C4^i^—C5—H5A	109.8
Ni1—N1—H1N1	98.8 (16)	N1—C5—H5B	109.8
C3—N2—C4	112.55 (10)	C4^i^—C5—H5B	109.8
C3—N2—Ni1	114.93 (8)	H5A—C5—H5B	108.3
C4—N2—Ni1	105.98 (7)	O1—C11—O2	124.55 (11)
C3—N2—H1N2	109.8 (15)	O1—C11—C12	119.64 (10)
C4—N2—H1N2	105.3 (14)	O2—C11—C12	115.81 (10)
Ni1—N2—H1N2	107.8 (15)	C12^ii^—C12—C11	123.39 (13)
N1—C1—C2	111.84 (10)	C12^ii^—C12—H12A	118.3
N1—C1—H1A	109.2	C11—C12—H12A	118.3
C2—C1—H1A	109.2	H1W2—O2W—H2W2	107.7
N1—C1—H1B	109.2	H1W3—O3W—H2W3	107.5
			
N2—Ni1—N1—C5	−166.65 (8)	O1W—Ni1—N2—C4	−106.83 (7)
N2^i^—Ni1—N1—C5	13.35 (8)	O1W^i^—Ni1—N2—C4	73.17 (7)
O1W—Ni1—N1—C5	104.78 (8)	C5—N1—C1—C2	179.36 (10)
O1W^i^—Ni1—N1—C5	−75.22 (8)	Ni1—N1—C1—C2	54.91 (12)
N2—Ni1—N1—C1	−39.09 (9)	N1—C1—C2—C3	−69.36 (15)
N2^i^—Ni1—N1—C1	140.91 (9)	C4—N2—C3—C2	−179.50 (10)
O1W—Ni1—N1—C1	−127.66 (8)	Ni1—N2—C3—C2	−58.06 (12)
O1W^i^—Ni1—N1—C1	52.34 (8)	C1—C2—C3—N2	71.78 (14)
N1^i^—Ni1—N2—C3	−139.74 (9)	C3—N2—C4—C5^i^	166.48 (10)
N1—Ni1—N2—C3	40.26 (9)	Ni1—N2—C4—C5^i^	40.06 (11)
O1W—Ni1—N2—C3	128.21 (9)	C1—N1—C5—C4^i^	−168.52 (10)
O1W^i^—Ni1—N2—C3	−51.79 (9)	Ni1—N1—C5—C4^i^	−38.86 (11)
N1^i^—Ni1—N2—C4	−14.78 (7)	O1—C11—C12—C12^ii^	11.2 (2)
N1—Ni1—N2—C4	165.22 (7)	O2—C11—C12—C12^ii^	−168.24 (16)

Symmetry codes: (i) −*x*+2, −*y*+1, −*z*; (ii) −*x*+1, −*y*, −*z*+1.

### Hydrogen-bond geometry (Å, °)

**Table:**

*D*—H···*A*	*D*—H	H···*A*	*D*···*A*	*D*—H···*A*
O1W—H1W1···O3W	0.85	2.17	2.8047 (14)	131
O2W—H1W2···O2^iii^	0.85	1.98	2.7026 (14)	142
O2W—H2W2···O2^ii^	0.85	1.91	2.7000 (15)	154
O3W—H1W3···O1^iv^	0.85	1.96	2.7633 (14)	157
O3W—H2W3···O1	0.85	2.06	2.7968 (14)	144
N1—H1N1···O2W^v^	0.88 (2)	2.19 (2)	3.0153 (15)	154 (2)
N2—H1N2···O3W^v^	0.90 (2)	2.25 (2)	3.0769 (15)	153 (2)
C3—H3B···O1^i^	0.97	2.60	3.3850 (18)	138

Symmetry codes: (iii) *x*, *y*, *z*−1; (ii) −*x*+1, −*y*, −*z*+1; (iv) −*x*+1, −*y*+1, −*z*+1; (v) −*x*+1, −*y*+1, −*z*; (i) −*x*+2, −*y*+1, −*z*.
